# Mosaicplasty in the Treatment of Osteochondral Lesions of the Talus: A Report of Two Cases

**DOI:** 10.7759/cureus.54345

**Published:** 2024-02-17

**Authors:** Mohammed Barrached, Achraf Tebbaa El Hassali, El Mahjoubi Sohaib, Najib Abdeljaouad, Hicham Yacoubi

**Affiliations:** 1 Department of Traumatology and Orthopedics, Mohammed VI University Hospital, Faculty of Medicine and Pharmacy, Mohammed Ist University, Oujda, MAR

**Keywords:** mosaicplasty, osteochondral graft, osteochondral lesion, talus, ankle pain

## Abstract

Osteochondral damage to the talus is one of the most frequent causes of ankle pain. In contrast to other joints in the lower limb, osteochondral damage of the talus is often attributed to traumatic events. One option of treatment is mosaicplasty, which has proved to be a feasible choice for the treatment of osteochondral lesions of the talus; it has the potential to alleviate ankle pain and facilitate engagement in daily activities as well as sports.

We present two different cases of osteochondral lesions of the talus, illustrating how this pathology can present clinically. Both cases involve males with no notable pathological antecedents. The first was the victim of a traffic accident, the second was the victim of a sports accident; they were admitted for the management of chronic ankle pain unimproved by analgesic treatment. Radiological findings revealed a talus osteochondral lesion in both patients, treated with an osteochondral autograft from the homolateral knee. Both patients progressed well, with the resumption of daily activities and sports. The notable result of current research is that mosaicplasty has been shown to have good results in those with large osteochondral lesions who want to return to normal activity.

## Introduction

Osteochondral damage to the talus is one of the most frequent causes of ankle pain [1-3], a condition common in young athletes following ankle injury [4]. In contrast to other joints in the lower limb, osteochondral damage of the talus is often attributed to traumatic events [5]. The effect of shear and compression forces leads to the bruising of cartilage and is frequently transmitted to the subchondral bone, leading to the occurrence of subchondral microfractures [6]. Besides trauma, contributing factors encompass endocrine or metabolic influences, genetic predisposition, vascular or synovial irregularities, localized elevated pressure, or persistent microtrauma [7].

There are a number of therapeutic techniques to choose from in treating osteochondral lesions of the talus; one option is traditional conservative non-operative treatment. Patients who are symptomatic have failed non-operative treatment or have acute avulsion debris should receive surgical treatment, including bone marrow stimulation, internal fixation, autologous bone or osteochondral grafting, and biological supplements for cartilage repair. The mosaicplasty has proven to be a feasible choice for the treatment of osteochondral lesions of the talus. In this presentation, we examine the impact of this therapeutic technique on the alleviation of symptoms in people with osteochondral lesions of the talus through the analysis of two case reports and a literature review.

## Case presentation

Case 1

A 20-year-old patient with no previous medical history, victim of a motorcyclist road accident, struck by a car with a point of impact on the right ankle. He was initially treated for an ankle sprain. However, five months later, the patient was referred to our department for a painful ankle unimproved by analgesics.

**Figure 1:**
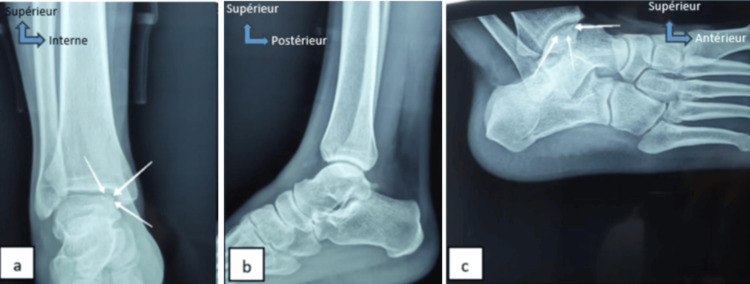
X-rays of the right ankle of case 1 (face (a), profile (b), 3/4 incidence (c)) shows osteochondral lesions of the medial part of the talus dome (white arrows).

Physical examination of the patient revealed a dodging limp on walking, pain on dorsiflexion, and palpation of the medial side of the right ankle. The rest of the physical examination revealed no other abnormal signs. Additional examinations, including ankle radiography and MRI, unveiled osteochondral lesions located in the medial portion of the talus dome (Figure 1). The patient underwent osteochondral autograft transplantation (mosaicplasty) from the homolateral knee. After 18 months, the mean postoperative American Orthopaedic Foot and Ankle Society (AOFAS) ankle score improved, pain on walking was absent and the patient resumed his daily activities. However, the pain emerged at the knee joint donor site for the patient, which was tolerated by the patient.

Case 2

A 35-year-old patient with no previous medical history, an occasional sportsman, was the victim of a sports accident resulting in trauma to his right ankle, initially untreated. Five months later, given the persistence of the pain, the patient consulted our department for therapeutic management.

**Figure 2:**
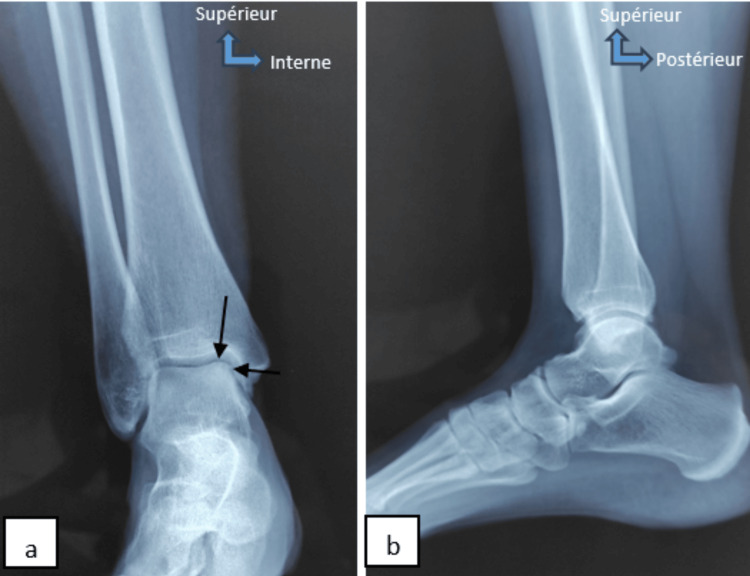
X-ray of the right ankle of case 2 (face (a), profile(b)) shows osteochondral lesions of the anteromedial part of the talus dome (black arrows).

Physical examination reveals normal gait, no limping, and ankle mobility pain, aggravated by plantar flexion. The rest of the physical examination revealed no other abnormal signs. Complementary examinations, including ankle radiography and MRI, unveiled osteochondral lesions located in the anteromedial portion of the talus dome (Figure 2). Like the first patient, the patient underwent osteochondral autograft transplantation (mosaicplasty) from the homolateral knee. After 12 months, the mean postoperative AOFAS score of the ankle improved, and the patient resumed his daily activities and sports. However, the patient did not develop pain in the donor site of the knee joint.

Surgical procedure

**Figure 3:**
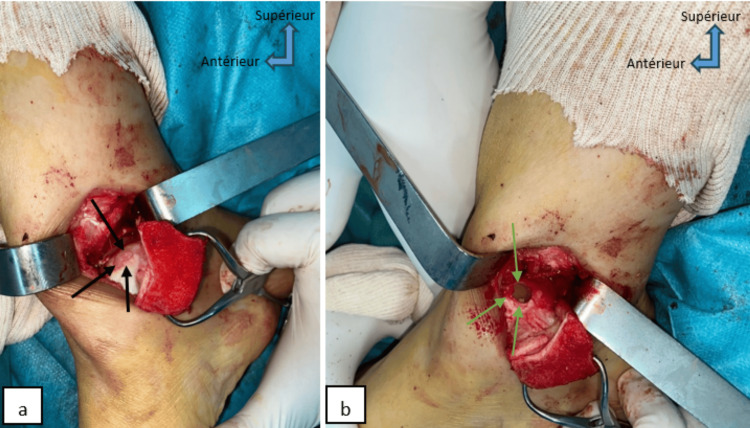
Perioperative image of case 2 shows medial malleolar osteotomy (a) for exposure of the anteromedial talar osteochondral lesion (black arrows), cleaning, and excision of the lesion (green arrows) (b).

**Figure 4:**
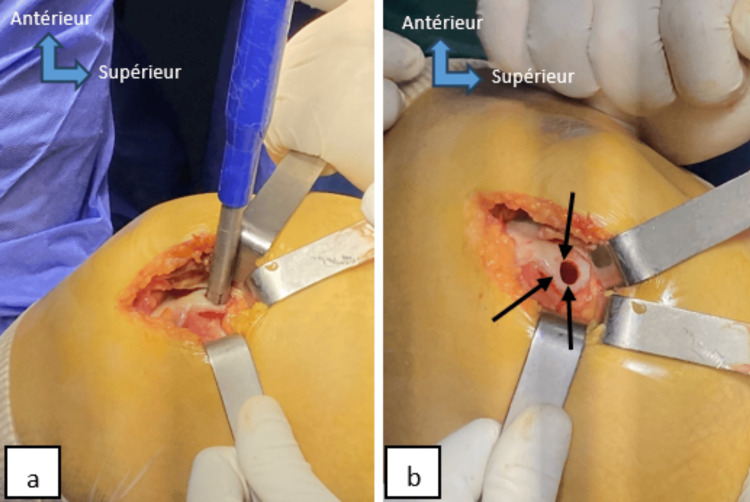
Perioperative image of case 2 shows lateral knee arthrotomy exposing the trochlear margin of the lateral femoral condyle, followed by removal of the osteochondral graft (a), graft harvesting site (black arrows) (b).

**Figure 5:**
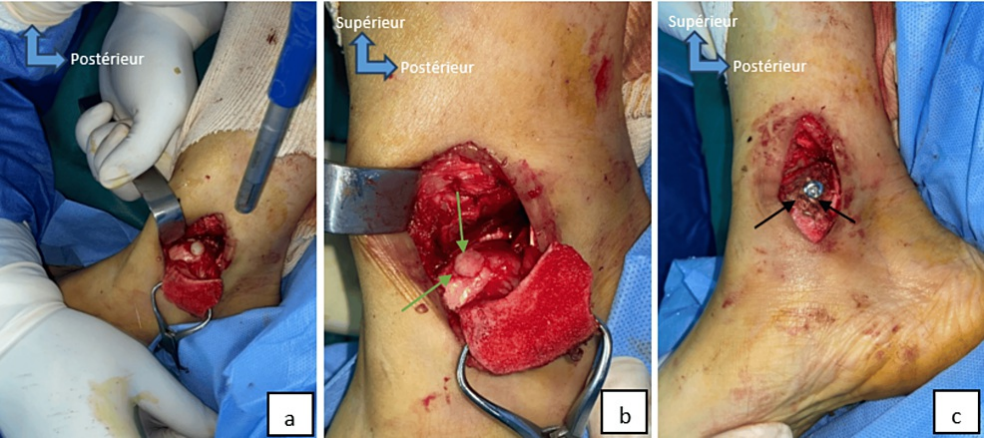
Perioperative image of case 2 shows the introduction of the graft into the recipient site (a), the final appearance of graft implantation (b) shows the surface of the osteochondral plug flush with the articular cartilage of the talus (green arrows), medial malleolus fixation hardware (black arrows) (c).

**Figure 6:**
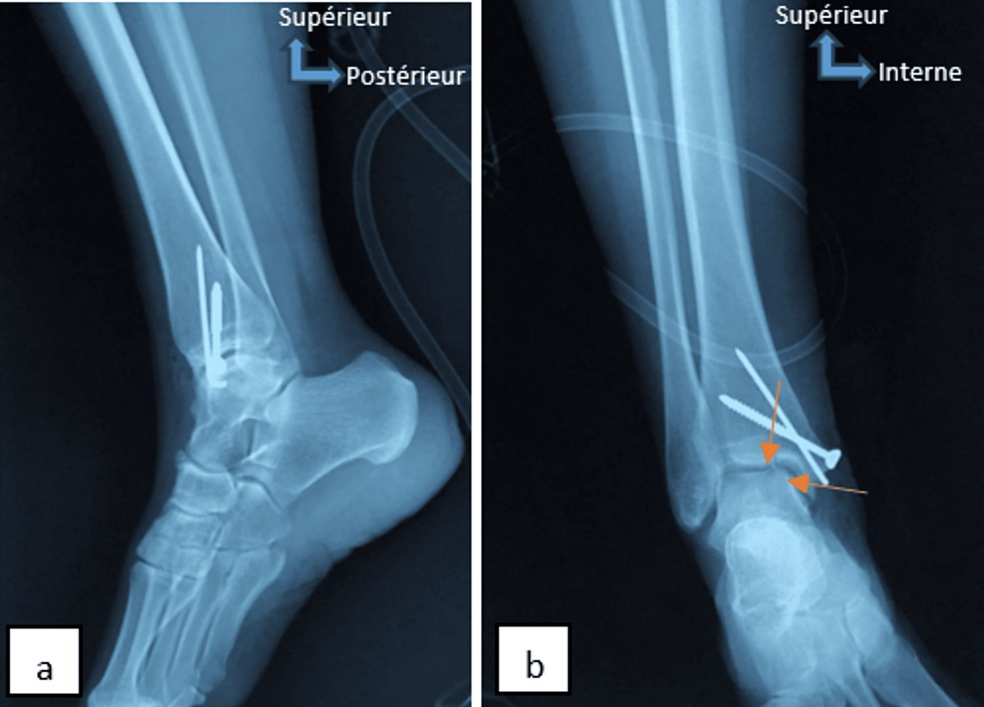
Postoperative control radiograph (face (a), profile (b)) shows medial malleolus fixation hardware and osteochondral graft in place (orange arrows).

Both patients received the same treatment protocol. Surgery was performed in dorsal recumbency, under spinal anesthesia, with a pneumatic tourniquet applied to the root of the limb. The first surgical step was to approach the osteochondral lesion of the talus, via the medial malleolus, a cutaneous and subcutaneous incision measuring four to five centimeters was performed, followed by an incision of the joint capsule at the front of the malleolus. A medial malleolar osteotomy was performed to overturn the bony mass of the medial malleolus and expose the medial lesion zone of the talus to identify the area of cartilage damage (Figure 3). Subsequently, the lesion was removed and thoroughly cleaned using a curette. Measurements of the recipient zone's length, width, and depth were taken and recorded. The second step of the operation involved harvesting the osteochondral graft. A lateral arthrotomy of the knee facilitated the extraction of grafts using a donor cutting tube from the non-weight-bearing surface of the lateral femoral condyle, employing the specific osteochondral autograft transfer system (Figure 4). The bone cylinder (osteochondral graft) was inserted, then the range of motion of the ankle joint was examined to ensure good stability of the graft (Figure 5). The two osteotomies were fixed with a cannulated screw and pin (Figure 6), followed by plane-by-plane closure of the approach and placement of a dressing.

Post-operative rehabilitation

Both patients benefited from functional rehabilitation of the ankle; the objective was to attain proprioceptive recovery, allowing the patient to resume their routine activities of daily living. The ankle remained unloaded for a duration of three weeks. Between weeks three and six, the patient underwent simple passive ankle movements and engaged in physical therapy. Partial weight-bearing exercises for the ankle joint commenced six weeks post-surgery, and full weight-bearing was permitted at eight weeks postoperatively.

## Discussion

Many patients experience a reduced quality of life due to symptomatic osteochondral damage of the talus. While the pathological mechanism is well comprehended, it is widely acknowledged that various etiological factors contribute to their origin [5]. It is also caused by malunion, degenerative arthritis, unusual lesions, and accessory protrusions or ossicles in this region [8]. Numerous procedures exist for addressing osteochondral damage of the talus, employing strategies such as cartilage repair, regeneration, and replacement [9,10]. Mosaicplasty is one of the treatment options for this disorder, it encompasses autologous grafting, utilizing one or more cylindrical components composed of both cartilage and its underlying bone; the components were obtained from a less weight-bearing section of the femur on the same side as the knee [11].

Our study demonstrated a significant improvement in patients' pain and function one year after mosaicplasty treatment. The results obtained in our cases reflect the findings of some other studies, including the example of 26 patients with type V osteochondral lesions of the talus treated with an osteochondral autograft, where the patients showed very satisfactory symptomatic improvement. Hangody et al. highlighted that this technique is among the methods yielding favorable long-term results [12]. Additional research has demonstrated varying degrees of symptomatic improvement, ranging from modest to excellent, in individuals with osteochondral damage of the talus who underwent mosaicplasty treatment [13,14]. On the other hand, Sexton et al. demonstrated in their study that mosaicplasty has a higher morbidity than alternative methods such as curettage. However, mosaicplasty offers significant advantages, notably the removal of necrotic subchondral tissue at the defect site [15].

The potential knee morbidity at the donor site may present challenges for patients; however, none of the published series have specifically addressed this aspect [16]. As a result, some authors propose the extraction of osteochondral plugs from the talus itself as a precautionary measure to prevent pain, stiffness, or potential arthritic changes in the knee, which could occur at the donor site [17]. In a retrospective analysis involving 11 patients, Reddy et al. showed that the clinical outcome was independent of the number of grafts acquired [18].

In general, as the size of the lesion increases, more sophisticated regeneration or replacement techniques are often necessary compared to those focused on simple repair [9]. Drawing upon literature findings and our own experiences, we can confirm that mosaicplasty has a positive impact on improving the condition of individuals with osteochondral damage of the ankle.

## Conclusions

The treatment of osteochondral lesions of the talus continues to improve, with studies demonstrating increasing clinical efficacy. Non-surgical therapy is typically preferred initially, with the goal of restoring patients' function and alleviating painful symptoms, thereby enabling them to resume their activities. Nevertheless, due to the uncertain ability of articular cartilage to heal effectively, non-surgical treatment is often of limited effectiveness at best, leading to a growing prevalence of operative interventions. On the basis of the available evidence and our own experience, autologous osteochondral grafting can be used as a primary treatment with good results. It can also be recommended as a second-line treatment when other techniques fail.

## References

[1] O'Loughlin PF, Heyworth BE, Kennedy JG (2010). Current concepts in the diagnosis and treatment of osteochondral lesions of the ankle. Am J Sports Med.

[2] Natsuhara KM, Sarcon A, Kreulen C (2019). Treatment of osteochondral lesions of talus with extracellular matrix cartilage allografts. Tech Foot Ankle Surg.

[3] Kim TY, Song SH, Baek JH, Hwang YG, Jeong BO (2019). Analysis of the changes in the clinical outcomes according to time after arthroscopic microfracture of osteochondral lesions of the talus. Foot Ankle Int.

[4] Verhagen RA, Struijs PA, Bossuyt PM, van Dijk CN (2003). Systematic review of treatment strategies for osteochondral defects of the talar dome. Foot Ankle Clin.

[5] Looze CA, Capo J, Ryan MK (2017). Evaluation and management of osteochondral lesions of the talus. Cartilage.

[6] Laffenêtre O (2010). Osteochondral lesions of the talus: current concept. Orthop Traumatol Surg Res.

[7] Mubarak SJ, Carroll NC (1979). Familial osteochondritis dissecans of the knee. Clin Orthop Relat Res.

[8] Eren Ogut (2022). The Stieda process of the talus: the anatomical knowledge and clinical significance of an overlooked protrusion. Bull Natl Res Cent 46.

[9] Steele JR, Dekker TJ, Federer AE, Liles JL, Adams SB, Easley ME (2023). Republication of "Osteochondral lesions of the talus: current concepts in diagnosis and treatment". Foot Ankle Orthop.

[10] DeSandis BA, Haleem AM, Sofka CM, O'Malley MJ, Drakos MC (2018). Arthroscopic treatment of osteochondral lesions of the talus using juvenile articular cartilage allograft and autologous bone marrow aspirate concentration. J Foot Ankle Surg.

[11] Buda R, Pilla F, Ruffilli A (2018). Arthroscopic bone marrowederived cell transplantation in osteochondral lesions of the talus: surgical procedure, technical tips, and results-award winner. J Am Acad Orthop Surg.

[12] Hangody L, Füles P (2003). Autologous osteochondral mosaicplasty for the treatment of full-thickness defects of weight-bearing joints: ten years of experimental and clinical experience. J Bone Joint Surg Am.

[13] Assenmacher JA, Kelikian AS, Gottlob C, Kodros S (2001). Arthroscopically assisted autologous osteochondral transplantation for osteochondral lesions of the talar dome: an MRI and clinical follow-up study. Foot Ankle Int.

[14] Valderrabano V, Leumann A, Rasch H, Egelhof T, Hintermann B, Pagenstert G (2009). Knee-to-ankle mosaicplasty for the treatment of osteochondral lesions of the ankle joint. Am J Sports Med.

[15] Sexton AT, Labib SA (2007). Osteochondral lesions of the talus: current opinions on diagnosis and management. Cur Opin Orthop.

[16] Mitchell ME, Giza E, Sullivan MR (2009). Cartilage transplantation techniques for talar cartilage lesions. J Am Acad Orthop Surg.

[17] Sammarco GJ, Makwana NK (2002). Treatment of talar osteochondral lesions using local osteochondral graft. Foot Ankle Int.

[18] Reddy S, Pedowitz DI, Parekh SG, Sennett BJ, Okereke E (2007). The morbidity associated with osteochondral harvest from asymptomatic knees for the treatment of osteochondral lesions of the talus. Am J Sports Med.

